# Development and Evaluation of Polymethacrylate-Based Ophthalmic Nanofiber Inserts Containing Dual Drug-Loaded Dorzolamide and Timolol: In Vivo Study in Rabbit’s Eye

**DOI:** 10.3390/biomedicines13010200

**Published:** 2025-01-15

**Authors:** Ahmad Karami, Shahla Mirzaeei, Leila Rezaei, Ali Nokhodchi

**Affiliations:** 1 Nano Drug Delivery Research Centre, Health Technology Institute, Kermanshah University of Medical Sciences, Kermanshah 6715847141, Iran; ahmadkarami202@gmail.com; 2 Student Research Committee, School of Pharmacy, Kermanshah University of Medical Sciences, Kermanshah 6715847141, Iran; 3 Pharmaceutical Sciences Research Center, Health Institute, Kermanshah University of Medical Sciences, Kermanshah 6715847141, Iran; 4 Pharmaceutical Sciences Research Center, Rahesh Daru Novine, Kermanshah 6715847141, Iran; 5 Department of Ophthalmology, Kermanshah University of Medical Sciences, Kermanshah 6715847141, Iran; leyla_rezaei60@kums.ac.ir; 6 School of Life Sciences, University of Sussex, Brighton BN1 9QG, UK

**Keywords:** glaucoma, ophthalmic nanofiber insert, controlled release, drug-loaded Dorzolamide and Timolol, fixed combination

## Abstract

**Background/objectives:** The aim of the study was to create a nanofiber insert incorporating Timolol (TIM) and Dorzolamide (DOR), targeting the management of glaucoma. This condition encompasses a variety of chronic, advancing ocular disorders typically associated with elevated intraocular pressure (IOP). **Methods:** The insert was made of Eudragite RL100 (EUD) polymer, a biocompatible material with high bioavailability, using the electrospinning method. The inserts were studied for morphology, drug–polymer interaction, physicochemical properties, and in vitro drug-release study. The pharmacokinetic properties of fibers were examined alongside consideration for irritation using a rabbit model and cell compatibility. **Results:** The results of the in vitro drug-release test showed retention and controlled release of both DOR/TIM over 80 h. Morphological examination demonstrated uniform nanofibers with mean diameters < 465 nm. The cell compatibility test showed a high percentage of cell survival, and none of the formulations irritated the rabbit’s eye. The Area Under the Curve (AUC0-72) for DOR and TIM in EDT formulations was approximately 3216.63 ± 63.25 µg·h/mL and 2598.89 ± 46.65 µg·h/mL, respectively, with Mean Residence Times (MRTs) of approximately 21.6 ± 0.19 h and 16.29 ± 6.44 h. **Conclusions:** Based on the results, the dual drug-loaded nanofiber preservative-free system can potentially be a suitable alternative to eye drops and can be used to reduce fluctuation and dose frequency.

## 1. Introduction

Glaucoma involves a group of progressive eye diseases that are caused by increased intraocular pressure (IOP). In a normal eye, an increase in pressure gradually damages the optic nerve, which leads to a lack of connection between the retina and the brain and, ultimately, an irreversible loss of vision in the long term [1,2]. Glaucoma drug therapy includes different drug groups such as prostaglandin analogs, beta-blockers, carbonic anhydrase inhibitors, adrenergic agonists, parasympathomimetics and Rho-kinase inhibitors [3].

TIM belongs to the group of beta-blockers that mainly inhibit beta-adrenergic receptors and reduce aqueous humor production in the eye [4]. DOR is the first topical carbonic anhydrase inhibitor drug that lowers IOP by decreasing the production of aqueous humor and is used in beta-blocker resistance and beta-blocker contraindication cases. In addition, studies have shown that the combination of TIM and DOR possesses greater effects on reducing IOP than the single drug therapy [1,5]. Furthermore, compared to unfixed dosage forms, using the DOR/TIM fixed combination can improve tolerability, patient compliance, retention time, and convenience. [6].

Topical ophthalmic dosage forms are the most common pharmaceutical products that possess convenience, non-invasiveness and low side effects [7]. Eye drops, the most common topical eye medicines, have some significant drawbacks, such as poor absorption, poor bioavailability, short drug retention time and low patient compliance due to frequent administration. These result from the unique anatomy and structure parts of the eye, such as tears, limited precorneal surface and blinking [8,9,10]. Studies have depicted that 60% of the active ingredient is removed within the first 2 min. In addition, all the active ingredients on the surface of the cornea are eliminated in 15–25 min after using eye drops [11].

Nanotechnology innovations have led to new drug delivery systems that provide beneficial characteristics such as high tissue compatibility, low immunogenicity, low toxicity, easy penetration of the cornea and improved patient adherence due to less frequent use [8,12]. In comparison to eye drops, eye inserts offer a longer shelf life, reduced toxicity, fewer side effects and more accurate dosing. However, due to their solid structure, they may cause lacrimation and discomfort in the eyes [13]. One of the purposes of utilizing these systems is to create drug forms with controlled and sustained release patterns that could raise the bioavailability and resident time of the drugs [1,14]. A nanofiber-based drug delivery system is a novel method of drug delivery that has features such as being flexible and having a high length-to-diameter ratio with the potential for drug-controlled release [15,16].

It has been noted that electrospinning is the only method used to manufacture nanofibers on a large scale [17]. Nanofibers and microfibers are fabricated from solutions of polymeric materials, ceramics or composite solutions consisting of polymer nanoparticles as well as molten materials by electrospinning [18,19]. Nanofibers have been studied for ocular drug delivery due to their desirable properties such as cost-efficiency, high drug-loading capacity [20], high porosity and large surface area [21]. Also, it has been shown that the loaded drugs could be released in a controlled manner [22], which reduces the number of doses and improves patient acceptance [23]. Ophthalmic nanofiber inserts have been applied to sustained release delivery variety of drugs such as dexamethasone, gentamicin/methylprednisolone, azithromycin and triamcinolone [16,24,25,26].

The purpose of this paper was to design EUD nanofibers loaded with TIM and DOR by using the electrospinning method to attain a prolonged release profile and retention time in the eye. Following the evaluation and optimization of physicochemical characteristics, optimized nanofibers were evaluated in terms of in vitro‚ in vivo release studies and biocompatibility.

## 2. Materials and Methods

### 2.1. Materials

DOR and TIM were purchased from Sina Darou Inc. (Tehran, Iran). Eudragit RL100 (EUD) was purchased by Evonik Industries (Darmstadt, Germany). Fetal bovine serum (FBS) was purchased from Gibco (Carlsbad, CA, USA). The trypsin, 3-(4,5-dimethylthiazol-2-yl)-2,5-diphenyl-2H-tetrazolium bromide (MTT) and Dulbecco’s modified Eagle’s medium (DMEM) were also obtained from Sigma Aldrich (Darmstadt, Germany). Methanol, acetonitrile, acetone, trimethylamine, sodium hydroxide, fluid thioglycollate medium, soybean-casein digest agar medium and sabrodextrose agar were purchased from Merck (Darmstadt, Germany). All materials and reagents used were of analytical grade.

### 2.2. Fabrication of Nanofibers

Three polymer/drug formulation systems, including 10% EUD + 15% DOR (ED), 10% EUD + 10% TIM (ET) and EDT, were fabricated by electrospinning technique. To prepare a polymeric solution to make nanofibers, EUD was dissolved in methanol/acetone, 8:2 ratios with TIM and DOR separately. A customized two-nozzle electrospinning system was used. For the production of ED nanofiber, both syringes contained 10% EUD + 15% DOR, and for ET nanofiber, both syringes contained 10% EUD + 10% TIM. For the preparation of EDT nanofiber, one syringe contained 10% EUD + 10% TIM solution, and another syringe contained 10% EUD + 15% DOR solution. All nanofibers were prepared in the same electrospinning condition. The polymer/drug solution was introduced into a rotary collector that was covered in aluminum foil by injecting it into an injector using a polyethylene needle with an internal diameter of 0.1 mm at a flow rate of 0.1 mL/h. Between the injector and collector, a high voltage of 15 kV was applied. All processes were performed at 25 °C.

### 2.3. Evaluation of Physicochemical Characterization of Nanofibers

#### 2.3.1. Thickness

The thickness of nanofibers in five different parts of nanofibers was measured by a digital micrometer (±0.001 mm), and the averages were reported.

#### 2.3.2. Folding Endurance

This test is designed to describe nanofibers’ flexibility and their resistance to tearing [27,28]. Equal-sized pieces of nanofibers were folded at one point separately till they were torn or broken. The average number of successful folds from three replicates was calculated and reported.

#### 2.3.3. Moisture Uptake and Loss

A certain amount of nanofibers (25 mg) was introduced into a desiccator containing a saturated solution of aluminum chloride to produce 79.5% relative humidity inside the desiccator to conduct a moisture absorption test and anhydrous calcium chloride for moisture loss test. The nanofibers were weighed again after 72 h, and the percentage of weight change was reported.

#### 2.3.4. Swelling Degree

The release behavior of nanofiber is related to the swelling degree substantially. A certain amount (*W_f_*) of nanofibers were soaked in phosphate buffer solution (PBS) of pH = 7.4, and after 12 h, they were weighed again ($W_{s}$). The degree of swelling was calculated using the following equation:

$$\mathrm{Swelling}\;\mathrm{degree}\;\%=\frac{W_{s}-W_{f}}{W_{f}}\times 100$$

#### 2.3.5. Tensile Strength Test

To perform the tensile strength test, samples with dimensions of two centimeters in length and one centimeter in width were separated from nanofibers. In this test, the number of structural changes due to the application of external force in nanofibers is measured.

### 2.4. Contact Angle

Hydrophilicity and hydrophobicity of surfaces could be determined by contact angle test. In this experiment, an AM7815 Dino-Lite digital microscope (AnMo Electronics Corp., Taipei City, Taiwan) was used, and photos were taken at 500 millisecond intervals. One drop of deionized distilled water at 25 °C was poured on the nanofiber pieces. The whole process was recorded live. Image j version 1.52 software was used for analyzing captured pictures. In this experiment, three replications are considered for each sample.

### 2.5. Fourier Transform Infrared Spectroscopy (FT-IR)

The nanofibrous samples and powder samples were combined with micronized KBr powder. The mixture was subsequently pressed into tablets under 9 tons of pressure manually. By using an FT-IR spectrometer (Shimadzu IR PRESTIGE-21, Kyoto, Japan), the FT-IR spectra were obtained.

### 2.6. Surface Morphology

For surface morphology evaluation, field-emission scanning electron microscopy (FE-SEM, MIRA3, TESCAN company, Brno-kohoutovice, Czech Republic) was used. The samples underwent a gold coating process, and after drying the gold-coated samples using the vacuum chamber of THE SEM device, they were analyzed using an SEM. The accelerating voltage was set at 20–30 kV.

### 2.7. In Vitro Release Study and Release Kinetic

Each nanofiber, weighing 20 mg, was placed in a cellulose acetate membrane (Delchimica Scientific Glassware, cut off 12000, Milan, Italy) containing 250 μL of PBS of pH = 7.4 and then soaked in tubes containing 6 mL of PBS of pH = 7.4 separately. The medium was subsequently placed in a shaker incubator set at 37 °C and 100 rpm. To maintain the sink condition, the whole 6 milliliters of PBS was collected and replaced by fresh 6 mL 37 °C PBS of pH = 7.4 at specific times. The drug release quantification of EDT nanofibers was performed using High-performance liquid chromatography (HPLC) (Knauer-V7603, Knauer company, Berlin, Germany) at 253 nm for DOR and 293 nm for TIM concurrently. HPLC condition includes a C18 column as the stationary phase and acetonitrile: 1% triethylamine buffer (pH = 3.5) (5:95, *v*/*v*) as the mobile phase. The flow rate of the mobile phase was adjusted to 1 mL/min, and the temperature of the column was set at 37 °C. Consequently, the retention times for DOR and TIM were approximately 1.5 and 2.3 min, respectively.

The drug-release mechanism was evaluated by fitting in vitro release data to kinetic models, namely zero-order, first-order, Higuchi, and Korsmeyer–Peppas; the predominant release mechanism was determined by the model with the highest correlation and lowest mean percentage error (MPE%).

### 2.8. In Vivo Pharmacokinetic Study

The animal study was confirmed by the Ethics Committee of Kermanshah University of Medical Sciences (IR.KUMS.REC.1400.402). Six adult healthy New Zealand rabbits were utilized for evaluating of in vivo release study of EDT nanofiber. Separate restraining boxes were used for housing each rabbit. All animals have access to water and food freely.

The nanofiber pieces were sterilized by an Ultraviolet lamp. Six pieces of nanofibers, each weighing 20 mg, were prepared and individually placed in the cul de sac of the right eyes of each rabbit, while the left eyes were considered as control. At specific time intervals, 100 μL of sterile PBS buffer (pH = 7.4) were poured into the cul de sac of rabbit eyes, and after 20 s, tear samples were collected by the sampler. Then, samples were quantified by the same HPLC method detailed in Section 2.6 for the determination of drug concentration.

### 2.9. Irritation Test

Evaluation of ophthalmic tolerance of EDT nanofiber was performed based on our past study [24]. Briefly, a 20-mg piece of nanofiber was placed in the cul de sac of a healthy New Zealand rabbit eye, and the other eye was considered the control eye. In the specific time intervals (day 1, day 2 and day 3), eyes were observed according to THE Draize ocular irritation test parameter, including symptoms of damage to the iris, cornea and conjunctivae. Then, they were scored based on the alteration degree (0 = no alteration, 1 = mild alteration and 3 = obvious alteration).

### 2.10. Sterility Test

One of the important features of eye products is that they are sterile. Sterility means the absence of living microorganisms in the product. In order to maintain the sterility of nanofibers, materials and equipment were sterilized by autoclave at 121 °C for 15 min during the manufacturing process. In addition, the nanofibers were exposed to UV radiation for one hour. Three culture media, including sodium thioglycolate broth for anaerobic bacteria, soybean-casein digest broth medium for aerobic bacteria, and sabouraud dextrose broth for fungi, were used for the sterility test. The nanofibers were placed separately in three culture mediums, and then fluid thioglycolate and soybean-casein digest broth mediums were incubated in an incubator at 35.5 °C for 24 h. Sabrodextrose agar medium was incubated at 25 °C for a duration of 24 h. Positive and negative control samples were also prepared to compare the turbidity.

### 2.11. In Vitro Cell Cytotoxicity Test

Evaluation of EDT nanofiber cytotoxicity was performed according to our previous study [24]. Briefly, L929 (mouse fibroblast) cells were chosen and cultured in a 24-well plate and incubated for 48 h. One row of the plate was selected as the control and received none of the formulations. Pieces of one drug-loading nanofiber with certain weights were sectioned and submerged in 10 mL of Dulbecco’s modified Eagle’s medium (DMEM) to yield 50.0, 25.0 and 12.5 μg/mL concentrations of DOR and TIM, separately, in the medium. Then, 30 μL of MTT assay solution and 270 mL of Dulbecco’s modified Eagle’s medium (DMEM) containing 50.0, 25.0 and 12.5 μg/mL concentrations of DOR and TIM were added to the wells, followed by a 4 h incubation of plates. 150 μL of dimethyl sulfoxide (DMSO) solution was used for dilution of the solution after removal. To measure the cell viability, a microplate reader (GENios, Groedig, Austria) was used for the evaluation of the sample at 260 nm wavelength. The average result of three repeated tests was recorded, and the cell viability was determined using the subsequent equation:

$$Cell\;viability\left(\%\right)=\frac{Absorbance\;of\;sample}{Absorbance\;of\;control}\times 100$$

### 2.12. Statistical Analysis

To calculate the average of the measured values, each test was run three times. The statistical software SPSS 25.0 was utilized to perform a post-hoc Tukey’s test with a significance level of 0.05 to statistically compare the test results.

## 3. Results

### 3.1. Physicochemical Characterization of Nanofiber

**Figure 1:**
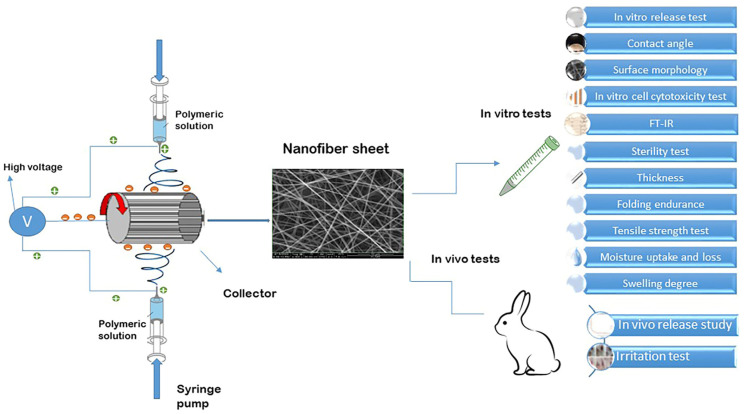
Schematic illustration of electrospinning procedure and tests conducted on designed nanofibers.

The pre-formulations were prepared through the modification of various variables, including electrospinning parameters, solvent composition, and polymer concentration. The refined formulations were then utilized for the fabrication of nanofibrous inserts loaded with drugs. Extensive research has been conducted on EUD due to its advantageous mechanical properties, gradual disintegration, biocompatibility, and lack of eye-related toxicity, especially within the realm of dual drug delivery applications for ophthalmic purposes [29]. The aim of this research was to create novel nanofibrous inserts for the continuous release of a dual therapy involving DOR and TIM to the eye. EUD was chosen as the base polymer and utilized in the electrospinning process to generate nanofibers loaded with both drugs due to their promising attributes. The electrospinning procedure and the tests carried out on the designed nanofibers are schematically depicted in Figure 1.

Thickness: The thickness of each nanofiber was measured at five different points. ED nanofiber was thinner than (0.128 ± 0.013 mm) ET and EDT nanofibers (0.129 ± 0.016 mm and 0.129 ± 0.0128 mm, respectively), but this difference was not significantly different. All nanofibers meet the criteria for thinness, making them suitable for insertion into the Cul de sac of the eyes without causing irritation.

Folding endurance: ED, ET and EDT nanofibers showed proper folding endurance. The range of folding endurance for nanofibers was found to be 161–171. These results indicated the convenient and easy flexibility of nanofiber in order to place in the eyes.

Moisture Uptake and Loss: The moisture uptake and loss percentage for all nanofibers was almost 1%. ET nanofiber showed the moisture uptake percent (1.004 ± 0.09), and ED nanofiber possessed the most moisture loss percent (0.91 ± 0.04). The results demonstrated the good stability of nanofibers in humid and dry conditions.

Swelling degree: All nanofibers indicated high swelling degree, and ED nanofiber showed the most swelling degree (192.42 ± 4.28%) compared to ET nanofiber (176.97 ± 3.72%) and EDT nanofiber (186.59 ± 4.37%). The reason for the high swelling degree of EUD nanofibers is related to the swellablity feature of EUD in water and to some extent, the hydrophilic properties of the mixed drug in the polymer. In 2013, Franca et al. designed bimatoprost-containing eye inserts. The swelling of the blank and drug-loaded inserts was reported to be 600% and 400%, respectively. An eye pressure study in rabbits showed that the use of blank inserts and drug-loaded inserts did not increase intraocular pressure compared to the untreated (control) condition, and the drug-loaded insert reduced intraocular pressure [30].

Tensile strength test: Ocular nanofiber should possess suitable strength and flexibility to maintain its integrity and non-irritancy to the eyes, respectively. ET nanofiber showed higher elongation % than other nanofibers.

The results of the physicochemical characterization test are shown in Table 1.

### 3.2. Contact Angle Results

**Figure 2:**
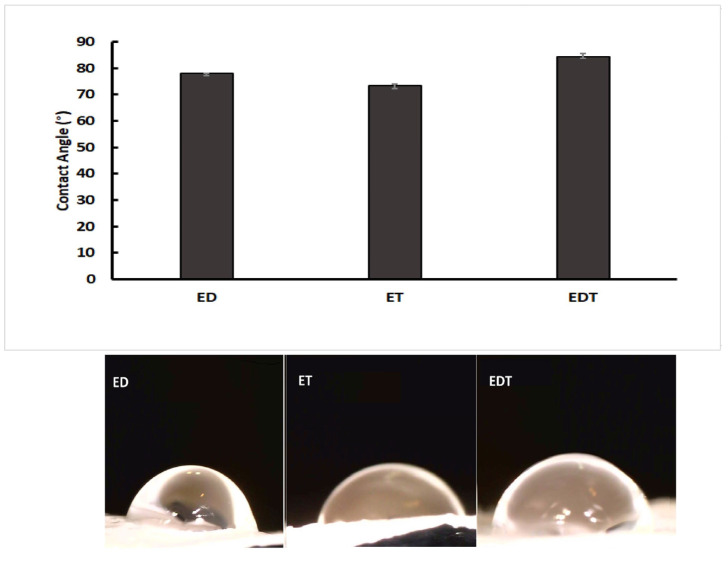
Comparison of contact angles measured for ED (DOR loading nanofiber), ET (TIM loading nanofiber) and EDT (DOR and TIM loading nanofiber). (Data is reported in mean ± SD, *n* = 3. *p*-Value > 0.05).

Based on Figure 2, when the contact angles of ET nanofibers were compared to EDT and ED nanofibers, it is evident that EDT nanofibers have a higher contact angle (84.44 ± 0.7 degrees) than ET nanofibers (74.24 ± 0.9) and ED (77.94 ± 0.7 degrees). The three nanofibers’ surfaces are wettable and hydrophilic because their contact angles range from 0 to 90 degrees. The difference in contact angles indicates that the hydrophilicity of EDT nanofibers is different from that of ET and ED nanofibers because of the presence of more hydrophilic drugs in EDT formulations.

### 3.3. Fourier-Transform Infrared Spectroscopy (FT-IR)

**Figure 3:**
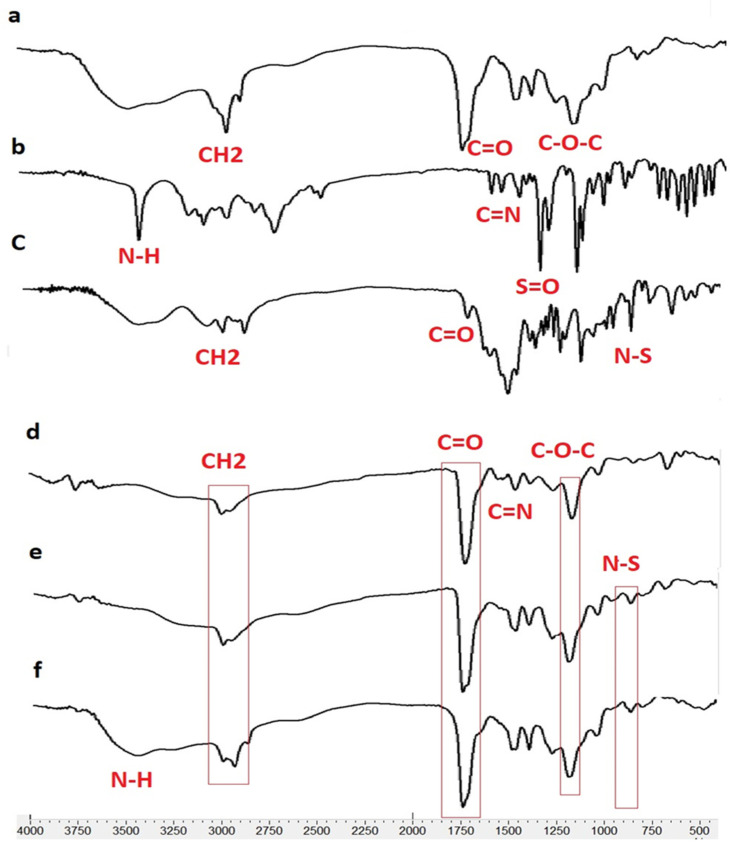
FTIR spectrum for Eudragit RL100 (**a**), DOR (**b**), TIM (**c**), ED (DOR-loading nanofiber), (**d**), ET (TIM loading nanofiber) (**e**) and EDT (DOR- and TIM-loading nanofiber) (**f**).

According to the FTIR results, the presence of TIM and DOR in the nanofibers was approved. In addition, the lack of a new peak in the FT-IR spectra of drug-containing nanofibers revealed that no new chemical compound was created during the nanofiber manufacturing process, nor were there any chemical reactions between the nanofiber’s components. In other words, there are only physical interactions between the components of drug-containing nanofibers. In the EDT nanofiber FTIR spectrum (Figure 3), peak 1730 cm^−1^ is related to the stretching vibration of the C=O ester bond, peak 1176 cm^−1^ is related to the stretching vibration of the C-O ester bond, and peak 2983 cm^−1^ is related to the stretching vibration of the C-H bond of EUD.

In the EUD-TIM-DOR nanofiber spectrum, in addition to the peaks related to EUD, peaks related to DOR are observed with the following specifications.

The peak around 650 cm^−1^ corresponds to the rocking vibration of the C-H bond, the peak around 1076 cm^−1^ corresponds to the stretching vibration of the SO_2_ bonds, and several peaks between 1020 and 1250 cm^−1^ correspond to the C-N bond. Furthermore, the peaks with the following specifications are related to the TIM in nanofiber.

Peaks 3253 and 3421 cm^−1^ are related to the stretching vibrations of NH and OH groups, peaks between 2850 to 2990 cm^−1^ are related to the bending vibration of the CH bond, peak 1654 cm^−1^ is related to the stretching vibration of the C=N bond, and 960 cm^−1^ peak related to N-S bond.

### 3.4. Surface Morphology

**Figure 4:**
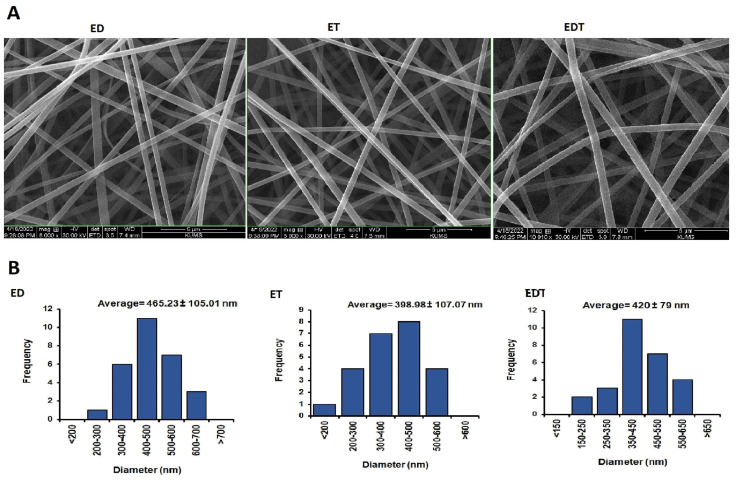
(**A**) SEM images of nanofibers at 8000× magnification for ED and ET and at 10,000× magnification for EDT. (**B**) Histogram diagram of nanofibers size distribution in nanometers. Data is presented in mean ± SD.

Nanofiber diameter and morphology were determined using SEM and Image J software (Figure 4). The results indicated that the average diameter of nanofiber filaments is 398–465 mm. It was shown that ED nanofibers had the highest average diameter among the nanofibers. This difference may stem from the viscosity properties of the polymer-drug blend in the solvent system, which influences the nanofiber production in the electrospinning technique. According to SEM images, all nanofibers have smooth, uniform and grain-free surfaces because the variables of both the solvent and polymer system and the nanofiber manufacturing process are optimized.

### 3.5. In Vitro Release Study and Release Kinetic

**Figure 5:**
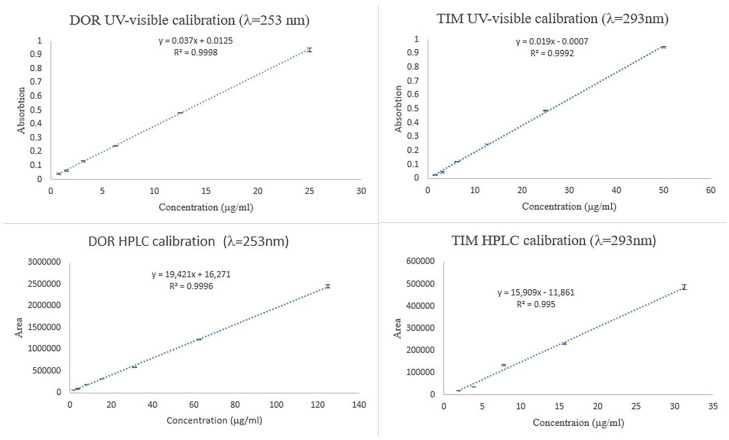
Calibration curve of TIM and DOR obtained by UV-visible and HPLC methods.

To investigate the concentration of TIM and DOR in ET and ED nanofibers, the UV-visible spectrophotometric method was used, and the calibration curve of the standard concentrations is shown in Figure 5. Also, to investigate the concentration of TIM and DOR in EDT nanofiber, the HPLC method was used, and the calibration curve of the standard concentrations is shown in Figure 5.

**Figure 6:**
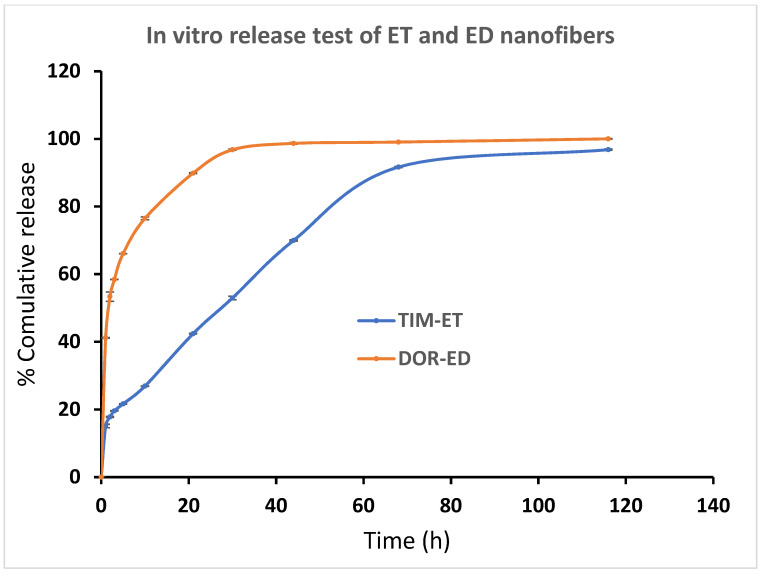
Cumulative percentage of DOR and TIM release from ED and ET nanofibers, respectively, in PBS buffer (pH = 7.4) at 37 °C. (Data is reported in mean ± SD, *n* = 3).

It was observed that loaded drugs released ED, ET and ED nanofibers during a long time period in vitro. A burst release pattern of drugs from formulations is followed by a controlled release pattern. According to Figure 6, around 41% of loaded DOR in ED nanofiber was released in the first hour and reached 76% in the next 9 h. This pattern was followed by slower speed and lasted for 80 h. Also, about 40% of DOR in EDT nanofiber has been released in 1 h, and 80% of DOR has been released in the next 9 h. Since then, the rest of the DOR experienced a slower controlled release in the 80 h (Figure 6).

**Figure 7:**
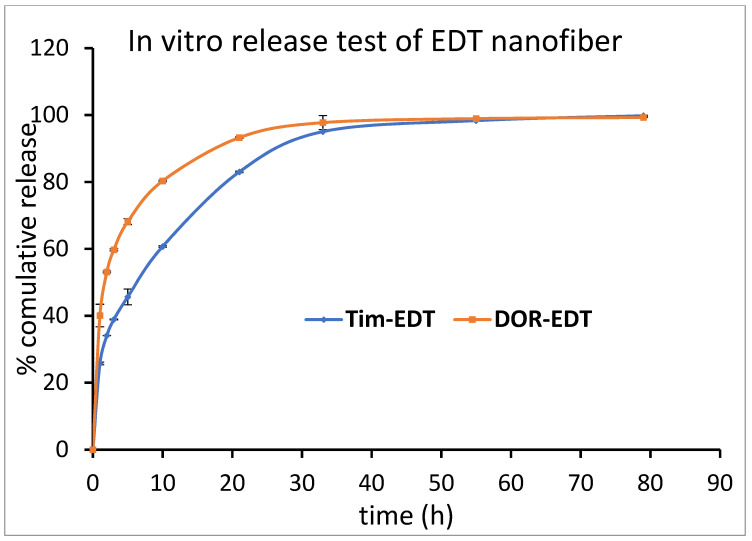
Cumulative percentage of DOR and TIM release from EDT nanofiber in PBS buffer (pH = 7.4) at 37 °C. (Data is reported in mean ± SD, *n* = 3).

ET and EDT nanofiber illustrated a significant controlled release pattern of TIM. Around 12% of loaded TIM in ET nanofiber was released in the first hour, and 26% of loaded TIM was released in the next 9 h. After 80 h, about 93% of the loaded drug was released in a controlled-released manner (Figure 6). There was a release of 21% of loaded Tim in EDT nanofiber in the first hour, and after 21 h, it reached 80%. Similar to the DOR release pattern, a slower controlled release pattern of TIM in 80 h was reported (Figure 7).

The three of the most ubiquitous, applied empirical models (Higuchi, zero order and First order) were chosen for the kinetic release comparison of nanofibers. The in vitro release data was matched in these models and the *R*^2^ was calculated for each one (Table 2).

As represented in Table 2, overall, all release data follow the Korsmeyer–Peppas model (higher R^2^ value with low MPE). The results showed that as the *n* value is less than 0.5, the main mechanism of drug release could be diffusion.

Based on the in vitro release test results, the incorporation of both drugs into EDT nanofiber as a dual drug delivery system did not significantly impact the drug release time when compared to ED and ET nanofibers used for single drug delivery (*p* > 0.05). The burst release pattern of the drugs may be related to the loaded drug on the surface of nanofibers. Nanofibers possess a high surface-to-volume ratio so that a great amount of the loaded drug in nanofiber can be in the outer layer of the nanofiber, and this phenomenon contributes to the burst release of drugs. This pattern of drugs could provide very quick therapeutic effects that could last for a long time, which has been followed by a controlled release pattern.

Due to the high permeability of EUD nanofibers, drugs can be diffused out from the core of the nanofiber, leading to a controlled release pattern. In simpler terms, the nanofibers’ high swelling and permeability are linked to the diffusion release mechanism.

### 3.6. Pharmacokinetic Study in Rabbit Model

**Figure 8:**
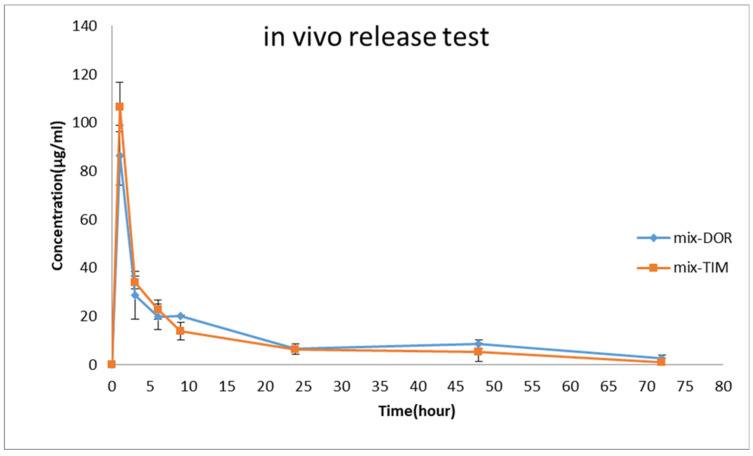
Tear concentration of DOR and TIM was measured after the administration of EDT to rabbits’ eyes during in vivo pharmacokinetics study (mean ± SD, *n* = 6).

Based on the findings of the in vitro release examination, the EDT nanofiber was selected for a subsequent in vivo release investigation. As illustrated in Figure 8, an immediate burst release profile is evident during the initial hour, succeeded by a sustained release profile spanning the subsequent 72 h. For TIM and DOR, the highest concentration was reached at 1 h, indicating a swift initial release that is essential for the loading dose. The maximum concentrations (C_max_) for DOR and TIM were 345.9 µg/mL and 426.1 µg/mL, respectively. Additionally, these formulations showed a prolonged release into the tear fluid, lasting up to 72 h, in which 0.93 µg/mL of TIM and 2.65 µg/mL of DOR were detectable in rabbit eyes at the endpoint. The Area Under the Curve (AUC) from the time zero to 72 h (AUC_0–72_) for DOR and TIM in EDT formulations was approximately 3216.63 ± 63.25 µg·h/mL and 2598.89 ± 46.65 µg·h/mL, respectively. Also, the Mean Residence Time (MRT) for these medications was calculated to be approximately 21.6 ± 0.19 and 16.29 ± 6.44. To conclude, the nanofiber inserts are marked by high AUC_0–72_ and MRT figures, owing to the effective drug encapsulation within the nanofibers, which ensures a stable and prolonged release from the nanostructure then it may keep IOP in the therapeutic and lower concentrations of the drugs.

The disparities observed between in vitro and in vivo release tests could be influenced by some limiting factors such as absorption, drainage, blinking and the sampling method.

### 3.7. Irritation Test

**Figure 9:**
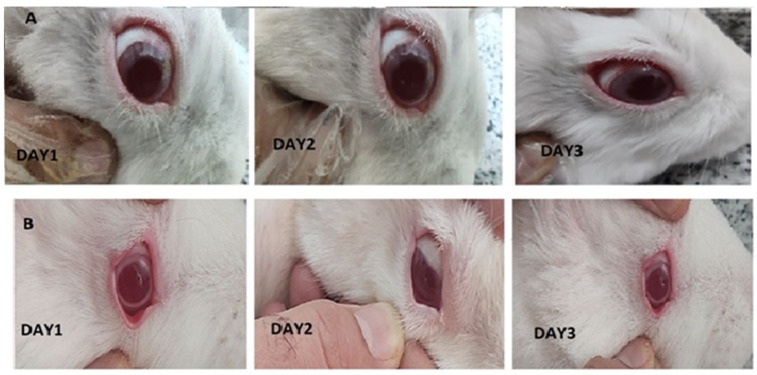
Images of rabbit eyes treated with a 20-miligram piece of nanofiber (**A**), control eye (**B**) based on the Draize test during irritation test on days 1 to 3.

As represented in Figure 9, there were not any differences between control and treated eyes. Indeed, redness, infection and continuous blinking were not detected. Examination of the conjunctiva (for chemosis, discharge, and redness), the cornea (for opacity and area of cornea involved), and the iris (for interruption for reaction to light) was conducted based on the Draize test on the scale of 0 to 3 scores. The observation revealed that in all cases, a score of zero was noted, indicating no eye irritation in the eyes of the rabbits.

### 3.8. Sterility Test

After 7, 14 and 21 days, the growth rate of microorganisms was evaluated based on the turbidity created in the culture medium. Samples containing sterile nanofibers and negative control showed no turbidity, while the positive control sample showed turbidity. As a result, it can be said that in the samples of nanofibers and negative control, no microbial growth has occurred and the sterilization process has been performed properly.

### 3.9. In Vitro Cell Cytotoxicity Test

**Figure 10:**
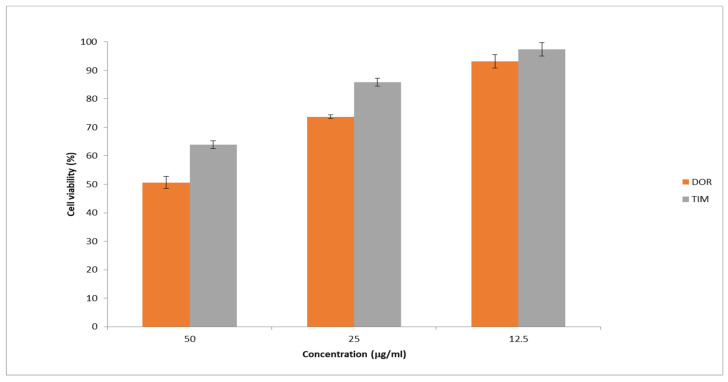
Cell viability was assessed using the MTT assay at different drug concentrations. The results are presented as the average value ± SD from three independent tests (*n* = 6).

Figure 10 presents the cell viability percentage against drug concentrations in samples. The obtained results showed the highest percentage of cell viability at the lowest drug concentration (12.5 mg/mL) was more than 90%. Moreover, the percentage of cell viability dropped with increasing drug concentration in the samples. In general, the obtained results exhibited that the cytotoxicity of prepared nanofibers is low, showing their potential as safe and effective carriers for drug delivery.

## 4. Discussion

Three ophthalmic nanofibers containing DOR and TIM alone or simultaneously were fabricated based on EUD polymer and using an electrospinning technique. In similar studies, such as the design and fabrication of ophthalmic nanofibers containing TIM [31], ophthalmic nanofibers containing triamcinolone [25], ophthalmic nanofibers containing TIM and DOR [27], the control group was not used in in vitro studies. In this study, the emphasis on in vitro tests was on the comparison between nanofibers containing a single drug and nanofibers containing both drugs and for this reason, the control group was not used in in vitro tests.

### 4.1. Physiochemical Tests

The nanofibers had sufficient flexibility and folding endurance to be separated from the collector and were easily separated from the aluminum foil. The results of the folding endurance test, a measure of the flexibility of nanofibers, showed that their folding endurance is 161–171 times and is more than the minimum desirable value of 40 times which has been reported in previous studies [29]. This indicates the appropriate flexibility of the designed nanofibers. The nanofibers have a thickness of approximately 0.130 mm. According to the results of previous studies, the optimal value of the thickness of nanofibers is approximately 0.4 mm, and in this range, the nanofibers are thick enough to maintain their integrity and also thin enough to provide comfort for use in the patient’s eyes [32]. The obtained results revealed that ET nanofiber has the highest tensile strength among nanofibers. One of the effective factors is the amount of loaded drug, and the lower the amount, the higher the tensile strength of nanofibers [33].

### 4.2. In Vitro Release Test

In previous studies, novel ophthalmic drug delivery systems such as nanoliposomes [34], inserts [35], proniosomal gels [36], in situ gel [37] and nanoparticles [38,39,40] have been utilized for the ophthalmic administration of DOR.

Kouchak et al. designed a nanoliposomal system containing DOR. In the in vitro release test, 70% of the loaded drug is released in the first hour in a burst release pattern with a subsequent slow release of the remaining drug over the following 6 h. Also, this drug delivery system has increased the ex vivo permeation of DOR compared to the drug solution after 8 and 24 h. In addition, the designed nanoliposomal system has reduced the IOP of rabbits over a period of eight hours [34].

Franca et al. examined the development of chitosan/hydroxyethyl cellulose ophthalmic inserts containing DOR by using a solvent/casting technique. It was observed that 75% of the drug content was released within the initial 3 h during the in vitro release experiment. Furthermore, the study of drug release in rabbit eyes showed that this formulation possesses the capability to release about 50% of the loaded drug [35]. Similarly, a study on designing nanoparticles containing DOR for ocular drug delivery revealed a release profile characterized by an initial burst release of 50% of the loaded drug within 2 h, succeeded by a gradual drug release over a duration of 8–10 h [38,39].

Also, nanoparticles have increased the transcorneal permeation of DOR compared to the drug solution [38,39]. Moreover, DOR-loaded nanoparticles have induced a more substantial reduction in the IOP of rabbit eyes in contrast to the DOR eye solution [40]. Proniosomal gels containing DOR, designed by Fuda et al., released 54.7–93.7% of the loaded drug within 8 h of the in vitro release test. Also, the results of comparing the IOP-reducing effect of this formulation with DOR eye drops (Trusopt) showed that this designed system could reduce IOP by 45.4 ± 8.2 in 8 h. In addition, the IOP-reducing effect of the designed system containing DOR continued for more than 8 h, but the IOP-reducing effect caused by the use of eye drops disappeared after 5 h [36].

The in situ gelling system containing DOR nanoparticles, designed by Katiyar et al., released 58% of the loaded drug in an in vitro release test within 8 h. Furthermore, this method of drug delivery exhibited enhanced transcorneal permeation of DOR in comparison to conventional eye drops, a phenomenon that may be attributed to the sustained and gradual drug liberation from this particular system [37]. In this study, DOR is initially released from the ED nanofiber during the in vitro release test, exhibiting a burst release pattern. Subsequently, a more prolonged, slower and controlled release of the loaded drug occurs. In comparison to the aforementioned mechanisms, the unique structure of the nanofiber resulted in a reduced initial drug release rate of 40% during the burst phase, with a controlled and gradual release continuing for a prolonged period lasting up to 80 h.

Drug delivery systems such as nanoparticles [41,42], in-situ gelling systems [43], nanofibers [31] and nanogels [44] have been used for the ocular delivery of TIM. Shokri et al. developed nanoparticles that released 70% of the loaded drug during the in vitro release test over a period of 25 h. In addition, these nanoparticles containing TIM reduced IOP within 24 h. In addition, compared to eye drops, the fluctuation of the average IOP has been reported to be less [41]. On the other hand, eye gel containing nanoparticles carrying TIM released 41% of the loaded drug within 24 h [42]. Another study focused on the nanogel formulated by Sojino et al. revealed that approximately 30% of the loaded TIM was released within 8 h during the in vitro release test. This particular drug delivery system also effectively decreased the IOP in rabbit eyes within 48 h [44].

On the other hand, the TIM-containing gelling system designed by Pakzad et al. led to an initial burst release of 40% of the encapsulated drug over 8 h, succeeded by a sustained release over the course of a week [43]. In our previous study, we designed a nanofiber containing TIM. The in vitro release experiments results showed the TIM was released within three days. The drug is released from the nanofiber in two stages: first, it bursts out of it, and then it releases in a controlled release pattern within 3 days. Also, investigating the effect of reducing IOP in the eyes of horses showed that nanofibers carrying TIM have the ability to reduce IOP for six days [31].

In 2014, Garg et al. designed nanofibers containing DOR and TIM using PVA and PCL polymers. The findings of the drug-release assessment conducted in vitro indicated the capacity of both nanofiber types to release medications within a span of 24 h. Investigating the effect of reducing IOP in rabbit eyes showed that the maximum effect of reducing IOP of PVA/PCL nanofibers was observed after 16 and 20 h, respectively. Additionally, these nanofibers showed the effect of reducing IOP for up to 72 h [27]. The method described in Section 2.7 was used to perform the in vitro release test. This method has also been used in other studies, such as the design and fabrication of ophthalmic nanofibers containing ketorolac [45], dental nanofibers containing alendronate [46], ophthalmic nanofibers containing TIM [31], etc. In this method, the nanofibers are always exposed to fresh buffer, which provides sink conditions for accurate investigation of the drug release rate. In this study, nanofibers have also been used for the simultaneous drug delivery of TIM and DOR. The outcomes of the drug release evaluation in vitro from EDT nanofiber unveiled that 40 and 80% of DOR were released from the nanofiber in 1 h and 10 h, respectively. Subsequently, the remaining drug was released at a slower rate over the following 70 h. Also, 21% and 80% of TIM were released from these nanofibers within 1 and 21 h, respectively, and then the rest of the drug was slowly released from the nanofibers till 72 h. Compared to previous studies, it provides a controlled release of drugs over an extended time frame. Hydrophobic Eudragit RL100 nanofibrous layers have demonstrated the capability to provide sustained and prolonged release of ofloxacin from ocular inserts for up to 95 h, as reported by S. Mirzaeei et al. in 2021. Achieving the therapeutic effect necessitates a controlled drug-release pattern, high retention within the ocular environment, and slow degradation rates. These essential characteristics are intrinsic to polycaprolactone (PCL) and Eudragit nanofibers, rendering them suitable for such applications [46].

As mentioned in Section 3.5, the results of the laboratory release test of EDT nanofibers were similar to ED and ET nanofibers, and the combination of DOR and TIM in a nanofiber had no significant effect on the drug release pattern compared to single drug nanofibers (*p* > 0.05).

### 4.3. In Vivo Release Test

Previous studies revealed that after topical administration of TIM solution, it is absorbed through aqueous humor, conjunctival epithelium, lacrimal channels and nasal mucosa from the eyes to the systemic circulation and leads to a reduction in its concentration in the cul de sac [47]. Also, stroma and cornea were reported to play important roles in the absorption of DOR after topical solution administration [48]. One significant limitation of this study is the unavailability of a tonometer, which prevented intraocular pressure measurement in the rabbits. Additionally, the absence of an appropriate animal model for pressure measurement further constrains the study. One of the most challenging parts of this research is to investigate the amount of drug released in the rabbit eyelids, which has been studied very little. To do this, the method described in Section 2.8, which is a very novel method, was used. This method has previously been used to measure drug concentrations in rabbit eyes in studies such as the design and fabrication of ketorolac ophthalmic nanofibers [45] and vancomycin/amikacin ophthalmic nanofibers [49]. It is worth mentioning that in this study, the in vivo drug release test from EDT nanofibers in the eyes of rabbits indicated that despite the mechanisms of drug absorption, both drugs could be detected in the cul de sac of rabbit’s eyes within three days, and the drugs, which have been released through nanofibers, were flowed out in a controlled manner within three days. In light of our research and study of present articles, this work is the first to detect TIM and DOR as a combination drug delivery system for 3 days in the cul de sac of the rabbit eye.

The pharmacokinetics of the drug was also investigated. Our study was limited by the lack of pharmacodynamics evaluation of this drug delivery system on intraocular pressure reduction. Furthermore, we could not measure the drug in different ocular tissues due to ethical constraints on euthanizing rabbits.

### 4.4. Drug-Release Mechanism

The controlled release pattern of both drugs was observed in both the in vitro and in vivo release evaluations of EDT nanofiber. The lacrimal system made the EUD polymer swell slowly, which made the in vivo release more gradual than the in vitro release.

Erosion and diffusion play key roles in the drug release process from nanofibers. The sudden release of drugs from nanofibers is usually attributed to drugs being released from the surface of the nanofiber. Then, the controlled release pattern of drugs is related to the hydrophilicity of the polymer and the drugs that continued in the form of erosion and diffusion [50]. Due to the structures, EUD has a high swelling degree and it leads to diffusion release mechanisms of drugs from EUD nanofibers [51,52]. This is in agreement with the *n* values reported for all formulations in Table 2 (*n <* 0.5), indicating the main mechanism of drug release from these nanofibers is diffusion.

## 5. Conclusions

The electrospinning method was utilized to prepare nanofibers containing TIM and DOR individually as well as in combination. EUD polymer was employed in the fabrication of these nanofibers, all of which exhibited favorable physicochemical stability and possessed smooth and uniform surfaces. In addition, the nanofibers demonstrated a desirable pattern of drug release. Notably, EDT nanofibers were found to sustain the release of the drugs within the rabbit’s eye for a duration of three days, offering an effective combination of both drugs. In contrast to the commercial 0.5% TIM/2% DOR eye drop, which necessitates twice-daily administration [6], this formulation requires fewer doses, thereby promoting patient adherence. Additionally, the absence of water in this formulation mitigates concerns related to microbial instability, and its preservative-free nature further enhances safety. The favorable tissue compatibility controlled and prolonged release observed in this formulation indicates its potential to serve as a viable alternative to traditional eye drops. Furthermore, the high potential for industrial production and market introduction underscores the promise of this drug delivery system.

## Figures and Tables

**Table 1:** Results of thickness, folding endurance, moisture uptake, moisture loss and swelling degree of nanofibers. Data is presented in mean ± SD. For all tests *p*-Value reported > 0.05.

Formulation/Test	ED	ET	EDT
Thickness	0.128 ± 0.013 (mm)	0.129 ± 0.016 (mm)	0.129 ± 0.0128 (mm)
Folding endurance	161 ± 2.16	167 ± 1.70	171 ± 1.63
Moisture uptake (%)	0.85 ± 0.07	1.004 ± 0.09	0.848 ± 0.51
Moisture loss (%)	0.91 ± 0.04	0.84 ± 0.80	0.618 ± 0.03
Swelling degree (%)	192.42 ± 4.28	176.97 ± 3.72	186.59 ± 4.37
Tensile strength (MPa)	1.445 ± 0.79	2.18 ± 0.45	1.08 ± 0.83
Elongation at Break (%)	3.884 ± 0.76	12.86825 ± 2.07	3.1722 ± 0.23

**Table 2:** The $R^{2}$ of kinetic models for nanofibers.

Drug Formulation	Higuchi	Zero-Order	First-Order	Korsmeyer–Peppas
TIM-ET	0.969 (10) *	0.899 (18)	0.977 (21)	0.948 (8) *n* = 0.36 **
TIM-EDT	0.756 (12)	0.964 (23)	0.995 (2)	0.995 (1) *n* = 0.36
DOR-ED	0.876 (9)	0.705 (15)	0.932 (8)	0.985 (2) *n* = 0.33
DOR-EDT	0.812 (11)	0.626 (16)	0.912 (12)	0.993 (1) *n* = 0.36

* Numbers inside the parenthesis are mean percentage error (MPE%). ** *n* value is an indication of the mechanism of drug release; *n* < 0.5 diffusion, *n* = 1 erosion.

## Data Availability

The data will be sent to applicants after their request from the corresponding author. The candidates must agree to a data access contract.
